# Molecular Approaches to Sarcoma Therapy

**DOI:** 10.1080/13577140220127530

**Published:** 2002-03

**Authors:** R. J. Olsen, S. R. Tarantolo, S. H. Hinrichs

**Affiliations:** 1 Department of Pathology and Microbiology University of Nebraska Medical Center Omaha NE 69198–6495 USA; 2 Department of Internal Medicine Hematology and Oncology Section University of Nebraska Medical Center Omaha NE 69198–6495 USA

## Abstract

Soft tissue sarcomas comprise a heterogeneous group of aggressive tumors that have a relatively poor prognosis. Although conventional therapeutic regimens can effectively cytoreduce the overall tumor mass, they fail to consistently achieve a curative outcome. Alternative gene-based approaches that counteract the underlying neoplastic process by eliminating the clonal aberrations that potentiate malignant behavior have been proposed. As compared to the accumulation of gene alterations associated with epithelial carcinomas, sarcomas are frequently characterized by the unique presence of a single chromosomal translocation in each histological subtype. Similar to the Philadelphia chromosome associated with CML, these clonal abnormalities result in the fusion of two independent unrelated genes to generate a unique chimeric protein that displays aberrant activity believed to initiate cellular transformation. Secondary gene mutations may provide an additional growth advantage that further contributes to malignant progression. The recent clinical success of the tyrosine kinase inhibitor, STI571, suggests that therapeutic approaches specifically directed against essential survival factors in sarcoma cells may be effective. This review summarizes published approaches targeting a specific molecular mechanism associated with sarcomagenesis. The strategy and significance of published translational studies in six distinct areas are presented. These include: (1) the disruption of chimeric transcription factor activity; (2) inhibition of growth stimulatory post-translational modifications; (3) restoration of tumor suppressor function; (4) interference with angiogenesis; (5) induction of apoptotic pathways; and (6) introduction of toxic gene products. The potential for improving outcomes in sarcoma patients and the conceptual obstacles to be overcome are discussed.


*Sarcoma* (2002) **6**, 27–42

ORIGINAL ARTICLE

### Molecular approaches to sarcoma therapy

R.J. OLSEN[1], S.R. TARANTOLO[2] & S.H. HINRICHS[1]

[1]*Department of Pathology and Microbiology, University of Nebraska Medical Center, Omaha, NE 69198–6495, USA,* [2]*Department of Internal Medicine, Hematology and Oncology Section, University of Nebraska Medical Center, Omaha, NE 69198–6495, USA*

**Abstract**

Soft tissue sarcomas comprise a heterogeneous group of aggressive tumors that have a relatively poor prognosis. Although conventional therapeutic regimens can effectively cytoreduce the overall tumor mass, they fail to consistently achieve a curative outcome. Alternative gene-based approaches that counteract the underlying neoplastic process by eliminating the clonal aberrations that potentiate malignant behavior have been proposed. As compared to the accumulation of gene alterations associated with epithelial carcinomas, sarcomas are frequently characterized by the unique presence of a single chromosomal translocation in each histological subtype. Similar to the Philadelphia chromosome associated with CML, these clonal abnormalities result in the fusion of two independent unrelated genes to generate a unique chimeric protein that displays aberrant activity believed to initiate cellular transformation. Secondary gene mutations may provide an additional growth advantage that further contributes to malignant progression. The recent clinical success of the tyrosine kinase inhibitor, STI571, suggests that therapeutic approaches specifically directed against essential survival factors in sarcoma cells may be effective. This review summarizes published approaches targeting a specific molecular mechanism associated with sarcomagenesis. The strategy and significance of published translational studies in six distinct areas are presented. These include: (1) the disruption of chimeric transcription factor activity; (2) inhibition of growth stimulatory post-translational modifications; (3) restoration of tumor suppressor function; (4) interference with angiogenesis; (5) induction of apoptotic pathways; and (6) introduction of toxic gene products. The potential for improving outcomes in sarcoma patients and the conceptual obstacles to be overcome are discussed.

**Key words:** *sarcoma, translocation, fusion protein, molecular therapy*

### Introduction

Sarcomas are non-epithelial tumors derived from embryonic mesoderm or neuroectoderm, and may arise from the peripheral nervous system, cartilage, bone, muscle, adipose tissue, vascular components and various fibrous structures of the body. Approximately 2600 new cases of bone and cartilaginous tumors are diagnosed each year in the United States and are commonly distinguished from soft tissue sarcomas that account for 5200 new cases each year.[1–5] Since there are multiple distinctive subtypes, each category of tumor is relatively uncommon compared to epithelial tumors such as breast or lung cancer. None-the-less, important lessons have been learned by studying these relatively rare neoplasms. Over the past two decades, advances in medical research have appreciably expanded our understanding of sarcoma biology. As compared to the accumulation of mutations associated with epithelial carcinomas,[1] cytogenetic studies of sarcomas have revealed structural abnormalities associated with specific histological tumor types and aided the development of sensitive diagnostic techniques.[2,6,7] Characteristic gene aberrations found in sarcomas are shown in Table 1. Balanced chromosomal translocations frequently juxtapose two unrelated genes to form a novel tumor-associated transcript (Fig. 1). Commonly, the unrelated genes are transcription factors such as *TLS* (translocated in liposarcoma) which is fused to *CHOP* (C/EBP homology protein) in myxoid/round cell liposarcoma with the t(12;16)(q13;p11) translocation.[8] Characteristic chromosomal changes have proved useful in establishing specific tumor subtypes with prognostic significance, such as t(X;18)(p11.2;q11.2) that joins *SYT* (synovial sarcoma translocation protein) to either *SSX1* or *SSX2* (synovial sarcoma X chromosome breakpoint protein 1 or 2) in synovial sarcoma.[9] Tumors with the *SYT/SSX1* fusion demonstrate a significantly

Correspondence to: S.H. Hinrichs, Department of Pathology and Microbiology, University of Nebraska Medical Center, Omaha, NE 68198-6495, USA. Tel.: +1-402-559-4116; Fax: +1-402-559-4077; E-mail: shinrich@unmc.edu

1357–714X print/1369–1643 online/02/010027–16 © 2002 Taylor & Francis Ltd
DOI: 10.1080/1357714022012753 0

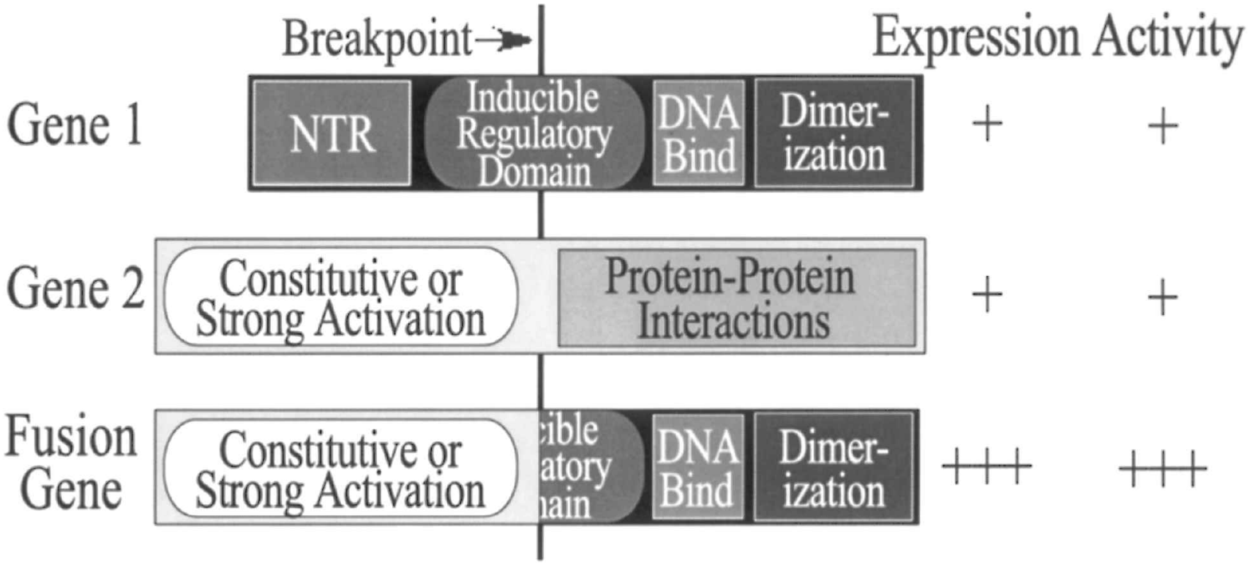

**Fig. 1.** *Schematic representation of modular gene components involved with chromosomal translocations. In this example, both genes are transcription factors and contain multiple components including a regulatory domain that may be silent or activated by specific signals, a DNA-binding domain and a dimerization domain. Exchange of genetic material at the breakpoint shown (arrow) results in a unique chimeric transcript that contains domains from both parental components. Due to this arrangement, the fusion protein may be over-expressed in tumor cells and demonstrate dysregulated activity. Although balanced translocations create two novel gene products, only one typically results in a functional protein associated with sarcomagenesis.*

**Table 1.** *Genetic mechanisms characteristic of human sarcoma*

| Tumor type | Molecular alteration | Involved genes |
|---|---|---|
| Alveolar | t(2;13)(q35;q14) | *PAX3/FKHR* |
| Rhabdomyosarcoma | t(1;13)(p36;q14) | *PAX7/FKHR* |
| Clear cell sarcoma | t(12;22)(q13;q12) | *EWS/ATF1* |
| Congenital fibrosarcoma | t(12;15)(p13;q25) | *TEL/NTRK3* |
| Dermatofibrosarcoma | t(17;22)(q22;q13) | *COL1A1/PDGFβ* |
| DSRCT | t(11;22)(p13;q12) | *EWS/WT1* |
| Extraskeletal myxoid | t(9;22)(q22;q12) | *EWS/TEC* |
| Chondrosarcoma | t(9;17)(q22;q12) | *TAF2N/TEC* |
| Embryonal | Trisomy 2 | Unknown |
| Rhabdomyosarcoma | Trisomy 8 | Unknown |
| Epithelioid sarcoma | LOH 22q | Unknown |
| Ewing's sarcoma | t(11;22)(q24;q12) | *EWS/FLI1* |
| | t(21;22)(q22;q12) | *EWS/ERG* |
| | t(7;22)(p22;q12) | *EWS/ETV1* |
| | t(2;22)(q33;q12) | *EWS/FEV* |
| | t(17;22)(q12;q12) | *EWS/E1AF* |
| | Trisomy 8 | N/A |
| | Trisomy 12 | N/A |
| Fibrosarcoma | t(12;15)(p13;q25) | *ETV6/NTRK3* |
| GI stromal tumor | 4q11–21 mutation | *c-KIT* |
| Hemangiopericytoma | t(12;19)(q13;q13) | Unknown |
| Leiomyosarcoma | t(12;14) | Unknown |
| Malignant fibrous histiocytoma | Complex karyotype | Unknown |
| Malignant rhabdoid tumor | del 22(q11.2) | Unknown |
| Myxoid/round cell | t(12;16)(q13;p11) | *TLS/CHOP* |
| Liposarcoma | t(12;22)(q13;q12) | *EWS/CHOP* |
| Synovial sarcoma | t(X;18)(p11;q11) | *SYT/SSX1* |
| | t(X;18)(p11;q11) | *SYT/SSX2* |

poorer prognosis than the *STY/SSX2* gene fusion.[10] Alternatively, sporadic mutations occur that secondarily influence neoplastic development by providing additional growth advantages.[11] In combination with biochemical analyses, these studies have provided significant insight into the molecular nature of sarcoma cells and their mechanism for neoplastic transformation.[12,13] It is generally believed that these characteristic chromosomal abnormalities represent the initiating genetic event and possibly constitute the underlying molecular mechanism for malignant proliferation. However, new treatment modalities exploiting this knowledge have only recently begun to appear.

The therapeutic management of soft tissue sarcomas represents a difficult clinical challenge. Aggressive surgical intervention provides the only effective means for a successful cure; however, many tumors are either too large or are located within vital structures and deep tissues that prevent their total resection.[5,14–19] Nearly 50% of all sarcomas recur within 2 years of treatment,[20] fewer than 25% of patients presenting with metastatic disease at diagnosis survive for 5 years[21,22] and the 5-year mortality rate following surgical failure is 91%.[23,24] The 1997 Sarcoma Meta-Analysis Collaborative Report on 1568 patients in 14 trials evaluating the potential impact of adjuvant chemotherapy agents clearly demonstrated the limitations of conventional therapy.[188] Local control and event-free survival for the 1568 enrolled patients was significantly improved by doxorubicin-based chemotherapy regimens; however, no benefits to overall survival were observed. Similarly, Gortzak *et al.* recently published the results of a randomized trial comparing neoadjuvant doxorubicin and ifosfamide plus surgery to surgery alone.[189] One hundred and thirty-four patients with high-risk sarcomas were evaluated with a median follow-up of 7.3 years. The 5-year disease-free survival and overall survival rates were similar in both groups. Disease-free survival was 56% in the chemotherapy plus surgery group compared to 52% in the surgery alone group, and the overall survival rates were 65 and 64%, respectively. Collectively, these studies suggest that adjuvant and/or neoadjuvant chemotherapy agents fail to significantly improve clinical outcomes and should not be routinely recommended for sarcoma patients. Thus, laboratory research directed towards the development of improved therapeutic options is clearly needed.

The goal of molecular therapeutics is to treat specific human disease with biomolecules engineered to operate at the genetic level. The underlying belief is that pathological processes can be alleviated by either altering gene expression, manipulating cellular activities or enhancing immune function. The embryological relationship between tumors arising from mesenchymal tissues including both hematological malignancies and sarcomas, may hold special significance. By targeting a specific neoplastic process leading to chronic myelogenous leukemia (CML) and sarcoma, phosphokinase inhibitors have recently demonstrated the feasibility of using pharmacological approaches based on molecular mechanisms for cancer therapy.[3] Studies have been expanded to include targeting of a second mesenchymal tumor type, gastrointestinal stromal tumor (GIST), with a tumor specific *c-KIT* (CD117) mutation.[27,28] However, this was not the first successful phamacomolecular approach as it had been preceeded by the use of all *trans*-retinoic acid for the treatment of acute promyelocytic leukemia (FAB M3) with a translocation of chromosomes 15 and 17.[25,26] Both improved response and outcome were achieved.

The poor clinical outcome and unique molecular basis of soft tissue sarcoma has stimulated numerous investigations into its susceptibility for gene-based approaches. These emerging strategies seek to eliminate tumor growth by utilizing molecular techniques that target the characteristic gene properties defining each tumor.[2] By specifically targeting these genetic changes and their downstream effects, the transformed phenotype may be eliminated. This review was initiated to identify published studies that have targeted mesenchymal malignancies arising from characteristic chromosomal aberrations and to expand the number of targets appropriate for further study. A number of promising new therapeutic strategies for sarcoma were found and are reviewed here, including disruption of chimeric transcription factor activity, inhibition of growth stimulatory post-translational modifications, restoration of tumor suppressor function, blockade of angiogenesis, induction of apoptotic pathways and introduction of toxic gene products. Molecular approaches using cell-mediated immunity (CMI) and tumor vaccination have been recently reviewed and are not discussed here.[14,29,30]

### Disruption of chimeric transcription factor activity

Cytogenetic studies have revealed a common theme of nature in which balanced translocations generate an aberrant transcription factor by joining the DNA binding domain of one protein to the potent activation domain of a heterologous partner. Transcription factors such as ATF1 and FLI1 are intimately associated with vital cellular processes and the effect of their incorporation into chimeric proteins is consistent with their role in regulating cellular proliferation and differentiation through control of gene expression.[40,41] In tumor cells, the resulting fusion protein tends to be over-expressed, displays dysregulated activity and demonstrates an increased transactivation and transforming potential in comparison to its native counterparts.[31–33,35] Transcription factors are typically composed of multiple domains having discreet functions such as DNA binding, dimerization or regulation; however, the combination of different domains from unrelated genes results in a chimeric protein having properties distinct from its individual components (Fig. 1). The dysregulated activation of target genes by fusion proteins has been hypothesized to be the primary initiating event in sarcomagenesis. For example, t(12;22)(q23;q12) joins *EWS* (Ewing's sarcoma protein) to *FLI1* (Friend leukemia virus integration site 1 protein) in

Ewing's sarcoma of bone and t(11;22)(q13;q12) fuses *EWS* to *ATF1* (activating transcription factor 1) in clear cell sarcoma of tendon sheath and aponeuroses.[38] Alveolar rhabdomyosarcoma is characterized by the translocation t(2;13)(q35;q14) that fuses the *PAX3* or *PAX7* and *FKHR* (Forkhead) transcription factors.[39] The clonal nature of each genetic abnormality and its consistent association with a specific tumor type reinforces its apparent importance to the underlying neoplastic program. The EWS/FLI1, PAX/FKHR and TLS/CHOP proteins associated with Ewing's sarcoma, alveolar rhabdomyosarcoma and myxoid liposarcoma, respectively, transform murine fibroblasts, whereas none of the separate genes composing the fusion are capable of transformation.[33–36] Since expression of the chimeric transcription factor may also play an important role in the continued progression of malignant behavior, disrupting its activity represents an attractive goal for the design of new therapeutic agents.[2,37]

Ewing's sarcoma, the second most frequently diagnosed childhood tumor of bone, represents an optimal choice for targeting due to its relatively high incidence and consistent association with the *EWS/FLI1* chimeric gene.[42] Ouchida *et al.* first suggested that fusion protein inhibition may be an appropriate strategy to sarcoma management.[43] Transfection of antisense plasmids reduced EWS/FLI1 expression and significantly impaired its transforming activity in murine fibroblasts. In addition, a 10-fold reduction in anchorage-independent colony formation in soft agar was observed, tumorigenicity in surgically prepared mice was blunted and susceptibility to etoposide and actinomycin D was increased.[35,44] Tanaka and associates reported that antisense oligonucleotides arrested the cell cycle at $G_0/G_1$, and the level of *in vitro* and *in vivo* cytotoxicity was related to antisense copy number.[44] No effect was observed in non-Ewing control cells. Kovar *et al.* demonstrated that interference with wildtype EWS/FLI1 function by a dominant negative *EWS/FLI1* derivative lacking the EWS transactivation domain effectively reduced the mitotic activity of cultured Ewing's sarcoma cells but had no effect on neuroblastoma cells.[45] Tumor growth was restored by overexpression of the wildtype *EWS/FLI* gene. Similar results have been independently reported by Toretsky *et al.*[46] and Lambert *et al.*[47]

Clear cell sarcoma, a rare tumor of tendon sheath and aponeuroses, is characterized by the EWS/ATF1 fusion protein.[48,49] Bosilevac and co-workers utilized a single-chain variable fragment (scFv) antibody termed scFv41.4 to interfere with the ATF1 basic DNA binding domain.[50,51] They hypothesized that it sterically interfered with the interaction between EWS/ATF1 and cyclic-AMP response element (CRE) containing promoters to inhibit transcriptional activation.[51] Intracellular expression of scFv41.4 in the human clear cell sarcoma cell line, SU-CCS-1, decreased endogenous EWS/ATF1 transcriptional activity in luciferase reporter assays and led to a significant induction of tumor cell death.[31] At 10 days post-transfection by retroviral vector, only 10% of SU-CCS-1 cells remained viable in Trypan blue dye exclusion assays and a 33% increase in the number of apoptotic nuclei was measured by flow cytometric analysis and TUNEL staining. No cytotoxic effect was observed in non-sarcoma control cell lines.

Alveolar rhabdomyosarcoma is the most common soft tissue sarcoma of children under 15 years of age and is a highly aggressive tumor arising from striated muscle.[52] Bernasconi *et al.* demonstrated that treatment of alveolar rhabdomyosarcoma cells with antisense oligonucleotides directed against the mRNA translational start site inhibited fusion protein expression and triggered an apoptotic cell death.[53] Similarly, Fredericks *et al.* have studied the ability of a dominant negative PAX3/KRAB transcriptional repressor to block the transforming activity of PAX3/FKHR.[54] The inhibitory molecule was shown to successfully compete with endogenous protein for PAX binding sites, and its expression resulted in a loss of cellular growth in low serum, reduced colony formation in soft agar and decreased tumor development in SCID mice.

### Post-translational modifications

One of the most important regulatory mechanisms of eukaryotic cells is the post-translational modification of proteins by phosphorylation. In addition, other post-translational modifications, such as glycosylation and prenylation, serve to control protein activity. The functional modifications may induce conformational changes that alter intermolecular interactions, stimulate downstream signal transduction cascades or dictate subcellular compartmentalization.[55,56] Since transcription factors are commonly composed of multiple domains with distinct activities, modifications of one component can allosterically influence the activity of another component. Several important studies have recently suggested that post-translational modification of sarcoma-associated proteins may represent an appropriate target for molecular intervention.

The insulin-like growth factor-1 receptor (IGF-1R), a transmembrane tyrosine kinase that modulates several tumor-associated activities including cellular proliferation, transformation and programmed cell death, is thought to play an important role in many different human sarcomas. It was recently observed that N-linked glycosylation was required for IGF-1R activity by stimulating transport to the cell surface. In support of the concept that blocking glycosylation may convey an anti-tumorigenic effect, Girnita *et al.* demonstrated that administration of tunicamycin and lovastatin to two Ewing's sarcoma cell lines resulted in the down-regulation of membrane-bound IGF-1R and a rapid decrease in cellular survival.[57] The growth arrest was later shown to be accompanied by reduced expression of the EWS/FLI1 fusion protein,[58] increased sensitization to doxorubicin cytotoxicity and enhanced susceptibility to exogenous apoptotic stimuli.[59]

The tyrosine kinase inhibitor STI571 has shown remarkable efficacy in treating chronic and accelerated phase CML and other human neoplasms associated with phosphorylated proteins.[60] Joensuu *et al.* recently published the report of a patient with rapidly progressive metastatic gastrointestinal stromal tumor (GIST) responding to STI571 treatment.[27] These mesenchymal neoplasms characteristically demonstrate dominant *c-KIT* (CD117) mutations that result in constitutive tyrosine kinase activity which may respond to inhibitors.[28,61] Over 11 months of treatment, the GIST patient underwent a significant clinical response.[27] No new lesions appeared, and the primary tumor regressed by 52%. Also, six of 28 liver metastases disappeared.[27] Similarly, STI571 was shown to significantly reduce the tyrosine kinase activity of the COL1A1/PDGFß (collagen 1A1/platelet-derived growth factor ß) fusion protein associated with dermatofibrosarcoma protuberans.[62] Treatment resulted in a loss of transforming activity *in vitro* and a reduction of tumor cell growth *in vivo*. STI571 has been well-tolerated with infrequent side effects,[63] and preliminary analysis demonstrated that 89% of GI stromal tumors unresponsive to standard chemotherapy showed a significant clinical response.[64] Based on these impressive results, independent phase I and phase II trials have been initiated for various sarcomas.

The EWS IQ domain, a conserved calmodulin-binding motif that contains an internal protein kinase C (PKC) phosphorylation site, is phosphorylated in tumor cells.[65] Presence of the IQ domain was hypothesized to regulate the transcriptional activation properties of EWS/ATF1 in clear cell sarcoma and EWS/FLI1 in Ewing's sarcoma. *In vitro* experiments demonstrated that intracellular phosphorylation of serine266 in the EWS IQ domain significantly enhanced the DNA-binding activity, transactivation potential and nuclear localization of both chimeric proteins. Furthermore, the clinical heterogeneity observed in some Ewing's sarcomas was theorized to be a function of post-translational modification since multiple fusion transcript variations involving different combinations of exons have been observed, and these have prognostic significance. Patients diagnosed with a Type I translocation involving *EWS* exons 1–7 and *FLI1* exons 6–9 have a better prognosis than all others; whereas tumors demonstrating a translocation that includes the additional portion of *EWS* containing the IQ phosphorylation domain are associated with a considerably poorer outcome.[66–68]

### Restoration of tumor suppressor function

Homozygous loss or mutation of the Rb tumor suppressor gene, such as occurs in retinoblastoma, is the classic example of a specific genetic change leading to malignant transformation. Mutations in the *Rb* tumor suppressor gene are observed in up to 70% of soft tissue sarcomas.[72,73] Whereas tumor suppressor loss may be a common early event in some sarcomas, in others, it may be an uncommon late event. Neoplastic growth and metastatic potential may be influenced by the abolition of tumor suppressor gene regulatory activity over cell cycle and apoptotic pathways. The presence of specific tumor suppressor gene mutations may confer a molecular profile that is predictive of tumor behavior and clinical response.[2,69] Numerous examples of targeted therapy for carcinoma focusing on restoration of tumor suppressor gene function exist in the literature. These reports have demonstrated the potential for using p53 pathways to reduce tumor burden at primary sites and limit metastases.[169] Accordingly, restoration of wild-type activity and reversal of the transformed phenotype by replacement of tumor suppressor genes has been proposed as a therapeutic strategy for sarcomas.[70,71] This is believed to be feasible since normal expression from only one of two genes may be sufficient to maintain wild-type function. Other tumor suppressor targets associated with various sarcomas are currently under investigation and include WT-1 (Wilm's tumor 1), MTS-1/p16 (multiple tumor suppressor 1) and RET (receptor of tyrosine kinase).[170]

A particularly high incidence of Rb gene mutations is reported in osteosarcoma, malignant fibrous histiocytoma, liposarcoma and mesenchymoma,[74] and these mutations strongly correlate with a poor overall prognosis.[75] Normally, Rb interacts with E2F to serve as a transcriptional repressor that inhibits cell cycle progression and controls the metabolic response to DNA damage. Any mutation that negatively alters Rb activity selects for increasingly aggressive clonal populations of transformed cells. Therefore, it was reasonable to propose that reintroduction of a normal *Rb* gene would have therapeutic benefit. Jiang *et al.* demonstrated that restoration of Rb protein to physiologically relevant levels in osteosarcoma cells resulted in a significant growth suppression *in vitro*.[76] Furthermore, transfection of constitutively active Rb constructs demonstrated an increased ability to arrest the cell cycle and inhibit tumorigenesis.[70]

A second well-studied tumor suppressor is the *p53* gene. Cytogenetic studies have revealed a significant incidence of *p53* alterations in many human cancers, including osteosarcoma, Ewing's sarcoma, malignant fibrous histiocytoma, leiomyosarcoma, liposarcoma and rhabdomyosarcoma.[11,74,77,78] In addition, patients with the familial Li-Fraumeni syndrome consisting of sarcomas, breast cancer, lymphoma, leukemia, adrenal and brain tumors have p53 abnormalities.[79] At the molecular level, wild-type p53 is a transcription factor that mediates cell cycle arrest, DNA repair and apoptosis.[80] Mutation is predictive of an increased tumor growth rate and reduced overall survival.[11] In many tumors, missense mutations lead to the expression of an inactive protein that either fails to bind DNA elements in p53-regulated promoters or activate their transcription. The ability of dominant-negative p53 to transform primary sarcoma cells *in vitro* and form clonal metastases in mice emphasizes its importance to the neoplastic process.[81,82] Accordingly, a number of novel approaches have been attempted to correct abnormal p53 function. Recent experiments demonstrated that p53 activity can be restored by the intracellular expression of an scFv that binds its negative regulatory domain.[71] Similarly, reintroduction of the wild-type gene to cultured leiomyosarcoma cells arrested the cell cycle[83] and resulted in a marked reduction of tumor development *in vivo*.[84] Comparable observations have also been recorded in models of osteosarcoma,[85] synovial sarcoma,[86] and alveolar rhabdomyosarcoma.[87] Furthermore, p53 expression has been shown to sensitize sarcoma cells to the cytotoxic effects of bleomycin, actinomycin D, 5-fluorouracil, doxorubicin, topotecan, etoposide and cisplatin.[87] Adenoviral introduction of chimeric tumor suppressor 1 (CTS1), a constitutively active p53 derivative that contains a truncated inhibitory domain and substituted VP16 activation domain, has also been demonstrated to retard osteosarcoma growth *in vitro* and inhibit tumor formation *in vivo*.[88]

Abnormal regulation of p53 and Rb in sarcomas may result from their interactions with proteins associated with double minutes. Double minutes are extrachromosomal circular DNA fragments often recognized on chromosome spreads of tumor cells. They may be present in large numbers and carry gene sequences that are consequently amplified in human cancer cells. The *MDM2* (murine double minute 2) proto-oncogene normally serves as a negative regulator of p53 and Rb by binding a regulatory domain that prevents their interaction with the CBP/p300 transcriptional adapter protein.[91,93] *MDM2* amplification has been observed in liposarcoma, malignant fibrous histiocytoma, alveolar rhabdomyosarcoma and osteosarcoma.[89–92] Since the corresponding loss of cell cycle control may be important to sarcomagenesis,[93,94] its restoration may represent an equally appropriate therapeutic strategy. Antisense-mediated inhibition of MDM2 in various soft tissue sarcoma cells restored p53 activity, decreased clonogenic survival *in vitro* and induced a dose-dependent apoptotic response *in vivo*.[58,95] Chemotherapy, particularly adriamycin and 10-hydroxycamptothecin, has additive or synergistic effects with antisense compounds targeting MDM2.[96]

### Inhibition of angiogenesis factors

A rich vascular supply is required to support the substantial metabolic demand of rapidly proliferating cells.[97] Solid tumors have been shown to stimulate angiogenesis by secreting factors that induce capillary recruitment, microvessel migration and endothelial expansion.[98] Folkman has hypothesized that the ability of transformed cells to activate vessel formation is fundamental to their continued maintenance of malignant behavior and any disruption in the supply of vital nutrients or removal of waste products could be detrimental to their potential for further metastatic growth.[99,100] Accordingly, many laboratories have begun studying the concept of neovascular inhibition as a therapeutic approach in sarcoma models.

The most frequent target of anti-angiogenesis strategies based on literature citations is *VEGF* (vascular endothelial growth factor). VEGF, a specific mitogen for endothelial cells *in vitro* and an angiogenic factor *in vivo*, is believed to serve as an essential survival factor for sarcoma.[101] In efforts toward achieving a therapeutic effect, several *in vitro* studies have attempted to disrupt VEGF activity with inhibitory antibodies,[102–104] small molecules[105] and antisense techniques,[106,107] and many clinical trials are now underway.[108,109] Zhang *et al.* have recently discovered that the p53 tumor suppressor gene exerts a negative regulatory effect on VEGF expression in several sarcoma cell types.[86] Their studies demonstrated that the angiogenic activity of wild-type synovial sarcoma cells was significantly lower in comparison to tumors expressing a mutant gene, and p53 restoration reduced the neovascularization and microvessel density of human leiomyosarcoma xenografts in SCID mice.[86]

The over-expression of *COX2*, an inducible isoform of cyclo-oxygenase, is believed to contribute to sarcomagenesis by inducing the synthesis of growth stimulatory prostaglandins. *COX2* expression is also associated with angiogenesis. Two recent reports independently demonstrated that specific COX2 inhibitors significantly decrease tumor growth in murine models of sarcoma.[110,111] Inhibition was also associated with a decreased level of tumor neovascularization and an enhanced cytotoxic sensitivity to irradiation.

Since angiogenesis relies upon the expansion of endothelial cell populations, an alternative therapeutic strategy is to directly target neovascular integrity. Recently, leiomyosarcoma transplants in athymic mice were effectively treated with an anti-endothelial cell monoclonal antibody.[112] $^{131}$I conjugation of the antibody further increased its inhibitory activity, evidenced by the presence of endothelial degeneration, capillary occlusion and tumor necrosis. In 1997, Ixsys Inc. initiated clinical trials with Vitaxin, a humanized MAb that recognizes $\alpha_v\beta_3$ receptors. These receptors are preferentially expressed on neovascular tissue, and antibody binding disrupts tumor-associated blood flow without disturbing the established vasculature of normal tissues.[113] Phase I dose escalation trials demonstrated that no significant toxicity was associated with Vitaxin treatment, and two-thirds of the enrolled patients experienced an objective clinical response.[114] Phase II trials were recently initiated at the M.D. Anderson Cancer Center with 15 leiomyosarcoma patients who were previously unresponsive to conventional treatment regimens, and additional protocols with other sarcoma types are planned.[114] A phase I trial reported clinical benefit in patients with advanced malignancies using SU668, a novel multi-receptor tyrosine kinase inhibitor, that acts on the VEGF (FLK-1), PDGF (PDGFR) and FGF-1 (FGFR) receptors.[3]

Kaposi's sarcoma is an unusual multi-centric vascular tumor associated with cellular immune deficiency and HIV infection. Essentially all patients with Kaposi's sarcoma are infected with human herpes virus 8 (HHV8), suggesting a prominent role in its pathogenesis.[115,116] In infected host cells, the virally encoded oncogenic G protein-coupled receptor (GPCR) constitutively promotes VEGF-mediated angiogenesis and spindle cell proliferation.[117] GPCR expression in fibroblasts was recently demonstrated to result in cellular transformation, and transfection of GPCR was shown to protect human umbilical vein endothelial cells (HUVECs) from the apoptotic response induced by serum deprivation.[118] Montaner and co-workers have suggested that GPCR signaling serves as a survival pathway for Kaposi's sarcoma cells and have begun efforts to elucidate potential mechanisms that may disrupt its tumorigenic effect.[118,119]

### Induction of apoptotic pathways

Neoplastic diseases are commonly characterized by the simultaneous presence of uncontrolled cellular proliferation and deficiencies in programmed cell death.[120] Apoptosis is normally regulated by a complex cascade of independent and interrelated signaling pathways. Any amplification of inhibitory genes or negative mutation of activator molecules can disrupt the delicate homeostatic balance between mitotic division, effector function and cellular turnover. The resulting loss of growth control has been shown to significantly influence sarcoma development.[37] Accordingly, new therapeutic approaches seeking to restore blocked apoptotic signals or exogenously induce programmed cell death are being investigated.

The *CD99/MIC2* gene encodes an integral transmembrane glycoprotein that is commonly present on mature lymphocytes and various undifferentiated cell lineages.[121,122] Although the molecular mechanism of its function remains largely undefined, CD99 has been implicated as a regulatory factor in cellular adhesion, proliferation, differentiation and apoptosis.[123–125] A particularly high density of CD99 molecules is observed on neuroectodermal progenitor cells, and its immunohistochemical detection has diagnostic value for peripheral neuroectodermal tumors (PNET).[126,127] The apparent restriction of CD99 to PNET and its distinct association with apoptotic pathways has led investigators to hypothesize that surface molecule engagement may have therapeutic potential. Sohn *et al.* first reported that *in vitro* cross-linking of CD99 by the agonistic DN16 monoclonal antibody induced apoptosis in Ewing's sarcoma cells, but had no effect on neuroblastoma cells.[128] Scotlandi *et al.* later demonstrated that ligation with the 0662 MAb also resulted in a significant inhibition of growth.[125] The resulting dose-dependent induction of apoptosis was absent in osteosarcoma control cells devoid of extracellular CD99. Furthermore, DN16 treatment reversed the transformed phenotype of Ewing's sarcoma *in vivo* and potentiated the cytotoxic activity of doxorubicin and vincristine. These preliminary studies suggest that CD99 engagement may translate into a highly specific therapeutic mechanism for Ewing's sarcoma. Since relatively high levels of CD99 are observed in a subset of malignant fibrous histiocytoma,[129] synovial sarcoma,[130] chondrosarcoma[131,132] and desmoplastic small round cell tumor,[133] the therapeutic potential of this technique may be extended to include a wide range of neuroectodermal tumors expressing the surface antigen.

The Fas/Fas-ligand system, also commonly termed Apo1/Apo1-L or CD95/CD95-L, is recognized as a major pathway for the regulated induction of programmed cell death. Fas-mediated down-regulation of lymphocyte populations and removal of autoreactive cells constitutes an important element to immune system development.[134,135] Recently, Fas expression in soft tissue tumors has prompted researchers to explore its potential as a molecular target in sarcoma. Kontny *et al.* first reported that all nine Ewing's sarcoma cell lines studied expressed Fas on their surface: three were readily killed by treatment with exogenous ligand, and four others underwent cell death following pre-incubation with interferon-γ (INF-γ) and cycloheximide.[136] Similarly, *in vitro* ligation with an agonistic monoclonal antibody induced a dose-dependent apoptotic response in 10 osteosarcoma cell lines.[137] The *in vivo* sensitivity of alveolar rhabdomyosarcoma, leiomyosarcoma and fibrosarcoma to these various Fas-mediated apoptotic pathways has also been confirmed.[138–140]

The nuclear factor-κB (NF-κB) family of eukaryotic transcription factors mediate expression of numerous genes having biological significance.[141] These include a variety of cytokines, interferons, growth factors and major histocompatibility complex (MHC) molecules. Activation has also been shown to confer protection against programmed cell death by suppressing pro-apoptotic pathways.[142] In the inactivated state, NF-κB dimerizes with I-κB, resulting in its cytoplasmic retention. However, upon stimulation, the complex dissociates and liberated NF-κB proteins enter the nucleus to interact with κB sites in target gene promoters. Recently, in a fibrosarcoma model, NF-κB inhibition by antisense oligonucleotides reduced cellular adhesion to extracellular matrices and colony formation in soft agar.[143] Similarly, transfection of a constitutive I-κB construct enhanced sarcoma cell susceptibility to tumor necrosis factor (TNF), daunorubicin and ionizing radiation,[96] and a dominant negative NF-κB gene reversed its inhibitory effect on apoptosis.[144] Human fibrosarcoma, leiomyosarcoma and malignant fibrous histiocytoma (MFH) cells also underwent a significant decrease in their viability as compared to null-vector infected cells, and *in vivo* treatment reduced tumor burden in SCID mice.

The transcription factor E2F is known to serve as a mediator of programmed cell death.[145] Hunt *et al.* reported that E2F activation in cultured leiomyosarcoma cells efficiently reduced their viability.[146] When wild-type E2F was administered to established transfectomas in nude mice, a marked inhibition of tumor growth was observed, and a fraction of the animals demonstrated complete regression.[147,148]

The basic fibroblast growth factor (bFGF) and FGF-receptor (FGF-R) occupy a critical role in the regulation of cell survival.[149] This system is particularly important during embryogenesis when cellular proliferation, differentiation and apoptosis must be tightly controlled. Sturla *et al.* hypothesized that bFGF and FGF-R may contribute to the initial development and physiological behavior of pediatric sarcoma.[150] Ewing's sarcoma cell lines that express bFGF and FGF-R were treated with exogenous ligand resulting in a dose-dependent decrease in cellular proliferation. Also, anchorage-independent growth of cells in soft agar and tumors in athymic mice were inhibited. Histological analyses of biopsy sections demonstrated a reduced cellularity and increased frequency of apoptotic nuclei following treatment.

Tumor necrosis factor-a (TNF-α) is a positive regulator of apoptosis. Although it mediates a death process that occurs independently of the Fas-ligand or NF-κB systems, their molecular pathways appear to overlap significantly.[142,151] TNF-α delivered by thermosensitive liposomes has been demonstrated to induce apoptosis in fibrosarcoma cells *in vitro* and implanted tumors *in vivo.*[152] Similarly, treatment with TNF-related apoptosis-inducing ligand (TRAIL) induced a cytotoxic response in Ewing's sarcoma.[153] The results of a large clinical trial spanning nearly 5 years and involving 186 patients in eight cancer centers were recently reported.[154] A major response was observed in patients with either an inoperable high-grade primary or recurrent sarcoma of the extremity. Treatment with TNF-α by isolated limb perfusion and tumor resection resulted in a 29% complete response rate and 53% partial response rate.[153]

### Toxic genes

A considerable obstacle to developing gene-based therapies is the successful targeting of every transformed cell in the tumor. Suicide gene therapy, also termed gene-directed enzyme prodrug therapy (GDEPT), holds an advantage over other techniques due to its ability to induce cytotoxicity in non-transfected cells. This bystander phenomena increases the effective dose of administered agents by exposing neighboring cells to toxic metabolites. Herpes simplex virus thymidine kinase (HSV-tk) is one example of the GDEPT system. When introduced into mammalian cells, tk efficiently phosphorylates the prodrug forms of ganciclovir (GANC) and acyclovir (ACV) into monophosphate nucleoside analogues that disrupt DNA synthesis, inhibit cellular proliferation and induce cytotoxicity. Early clinical experience with this approach in brain tumors demonstrated only short-term benefit.[155,156] Uchida *et al.* recently demonstrated that administration of GANC following retroviral transfection caused the necrotic death of human pelvic chondrosarcoma cells *in vitro* and inhibited the growth of chondrosarcoma tumors in nude mice.[157] Similar results have also been reported in murine models of fibrosarcoma,[158,159] glioblastoma[160] and osteosarcoma.[161]

The diphtheria toxin A chain (DT-A) has been studied as a potential suicide gene in sarcoma therapy. DT-A catalyzes the ADP ribosylation of cellular elongation factor 2 (EF2), resulting in the inhibition of mRNA translation and activation of apoptosis. Massuda *et al.* demonstrated that the selective expression of DT-A by a PAX3 responsive promoter induced cytotoxicity in PAX3/FKHR expressing alveolar rhabdomyosarcoma (ARMS) cells.[162] Tumors generated by murine sarcoma-180 cells have been shown to be eradicated by DT-A delivery.[163,164] Similarly, SN-38 (7-ethyl-10-hydroxycamptothecin), the toxic metabolite of CPT-11 (7-ethyl-10-(4-(1-piperidino)-1-piperdino)-carbonyloxycamptothecin), inhibits DNA repair by topoisomerase I and induces apoptosis following its activation by carboxylesterases. *In vitro* studies have demonstrated that human rhabdomyosarcoma cells stably transfected with rabbit liver carboxylesterase experience a significant increase in their sensitivity to CPT-11.[165,166]

### Discussion

The molecular strategies for sarcoma therapy reviewed here provide both concrete examples of progress as well as promising opportunities for the future. The essential observation is that clinical outcomes have been improved using rational therapeutic approaches directly targeting molecular pathways associated with cancer. Use of STI571 not only improves outcome in CML,[167] but it also caused regression in GI stromal tumor primary and metastatic lesions.[27] Future targets have been suggested from the functional disruption of chimeric transcription factors EWS/FLI1, EWS/ATF1 and PAX3/FKHR with reduction of tumor cell viability and induction of apoptosis *in vitro*.[10,65] It is not yet clear that molecular approaches can or should be the only therapeutic modality, and in fact, the combination of targeted therapy and conventional therapy including radiation and chemotherapy may be the best approach. Greater activity has been achieved with gene-based approaches when used in combination with multiple agents with a variety of toxic effects on cancer cells.[168]

Although a large number of recent scientific studies and publications have focused on the role of tumor-associated neovascularization in sarcomagenesis, immediate progress has not been forthcoming. However, significant obstacles and limitations to the use of anti-angiogenesis factors have been identified and, due to the discovery of these important mediators, substantial advances are expected to ensue.[100]

As might be expected in an emerging field, the number and variety of problems and limitations encountered is great. While the ability to disrupt specific pathways using molecular approaches has been clearly shown, the ability to deliver the potentially therapeutic molecule to the tumor remains problematic. Achieving intracellular accumulation of the therapeutic molecule is an even greater challenge. To be of medicinal value, the administered agent must possess two fundamental qualities. First, it must demonstrate great specificity in its tumoricidal activity. A drug that indiscriminately affects all cells will have little additional value in comparison to the currently available chemotherapeutic or radiation options. Second, it must be packaged in a manner that allows for delivery to the appropriate cellular target. Since gene-based drugs interact with specific molecular pathways, they are able to selectively confer cytotoxicity at relatively low doses. However, delivery mechanisms that effectively concentrate the pharmacological agent in tumor cells are not yet available. This is a particular concern for fusion proteins with transcriptional activity confined to the tumor cell nucleus. Extracellular epitopes can be easily accessed through vascular channels; however, the most attractive molecular targets under consideration are nuclear proteins. The inability to efficiently access the cellular compartments that contain chimeric transcription factors and tumor suppressor genes severely limits our capacity for testing molecular agents *in vivo*. Gene delivery represents the greatest obstacle to successfully applying these techniques in a clinical setting.[171] Advances in the drug delivery field should allow investigators to selectively transfect sarcoma cells by engineering viral vectors, immunoliposomes and high-affinity peptides that recognize sarcoma-associated surface antigens. Also, the continued development of restricted expression systems using inducible vectors and tissue-specific promoters will further enhance their therapeutic potential. Furthermore, vehicles that deliver these molecular agents to intracellular targets must be developed.

Recent experience from the monoclonal antibody field may be useful in predicting how progress is likely to be achieved in specific delivery of molecular therapeutics. When monoclonal antibodies (MAb) first appeared, much excitement was generated regarding their potential use in disease treatment, but few successes occurred beyond their use to deliver targeted radioactivity.[172] Now monoclonal antibodies that bind extracellular receptors have greatly impacted the treatment and survival of breast cancer, colon cancer, lymphoma and leukemia.[173] The potential for using the exquisite specificity of antibodies for selected epitopes has now been exploited to selectively kill diseased cells without disturbing healthy counterparts.[174] The clinical efficacy of Rituximab (anti-CD20) in non-Hodgkin's lymphoma, Campath-1H (anti-CD52) in chronic lymphocytic leukemia, Herceptin (anti-her2/neu) in breast cancer and Centuximab (anti-EGFR) in colorectal carcinoma has renewed interest in their potential application to soft tissue sarcoma.[175–178] One of the key advances in genetic engineering that made these successes possible was the ability to produce 'humanized' versions of murine monoclonal MAbs that minimize the immune response against rodent proteins due to species incompatibility. Recently, an anti-CD33 antibody (Gemtozumab ozogamicin) armed with a chemotherapeutic agent has been approved for acute myelogenous leukemia.[179] The antibody–drug combination is internalized and cleaved to deliver an intracellular dose of the active product. Addition of radioactive molecules to an antibody recognizing surface antigens has achieved killing of targeted cells as well as bystander cells without the surface receptor.[180,181]

Although MAbs provide an excellent tool for the engagement of surface antigens and soluble proteins, a majority of the nuclear targets identified for sarcoma intervention remain inaccessible since they are not naturally internalized. Recombinant antibody technology can circumvent this restriction by generating cloned MAb derivatives termed single chain variable fragments (scFv).[182] ScFv molecules are composed of an antibody heavy and light chain variable domain ($V_H$ and $V_L$) joined by a short peptide linker. Since its sequence is encoded by a single gene, the scFv cDNA can be introduced into cells and expressed as a functional protein having antigen-binding properties identical to its parental immunoglobulin. Also, the comparatively small size of scFv renders them relatively non-immunogenic to the host system and provides an advantageous pharmacological profile.[183,184] Previous studies have demonstrated that p53 tumor suppressor activity was restored by expression of an agonistic scFv targeting its regulatory domain,[71] and transcriptional activity of the EWS/ATF1 fusion protein was disrupted by an inhibitory fragment recognizing its DNA-binding domain.[31] The recombinant nature of scFv molecules also allows for their use in directing drug transport by incorporation into immuno-liposomes or viral coat proteins, and their activity can be enhanced by addition of leader sequences directing subcellular localization, high-valency multimer formation or increased tissue uptake and cellular retention.[185–187] Furthermore, when combined with structural studies and molecular modeling simulations, the fine specificity of antibody combining sites can be rationally engineered into bioactive protein loops or peptidomimetic compounds that display enhanced therapeutic effects.

Based on successes described here, the most fruitful targets for molecular intervention and additional investigation may be the post-translational modifications that potentially enhance chimeric transcription factor activity. In addition, targeting strategies should be investigated for other translocation-generated fusion proteins such as SYT/SSX in synovial sarcoma and TLS/CHOP in myxoid liposarcoma.[10,65] Experiments must determine whether the cytotoxic effects of inhibiting transcription factor binding are due to a reduced expression of growth stimulating genes or a downstream release from apoptotic inhibition. These concerted efforts will continue to expand our understanding of molecular sarcomagenesis and identify many new mediators and signaling pathways of potential importance. With this knowledge, we will be able to refine our current approaches and develop novel strategies that specifically counteract the neoplastic program at the genetic level.

Perhaps the greatest obstacle to overcome is the difficulty in obtaining sufficient resources to further develop therapies for tumor types that constitute less than 1% of all human cancers. However, the disproportionate number of cases that occur in children and the poor prognosis both argue for the necessity of continued effort. With adequate support, it is expected that many of the agents discussed here will become available for clinical evaluation, and their ability to improve patient outcomes will be critically evaluated. There remains significant concern that the true therapeutic potential of molecular techniques will not be immediately realized because of the way clinical trials are currently evaluated. The introduction of molecular therapy will require that response criteria applied to traditional cytoreductive chemotherapy strategies be reconsidered and significantly modified. Current measures of tumor response to therapy include complete remission (CR) or partial remission (PR), whereas stable disease (SD) is usually considered a treatment failure. However, improved performance status and stable disease may be an important measure of treatment outcome. Tumor response to molecular therapies should also be monitored with more sophisticated monitors of biological activity, such as real time PET scanning. In the future, cancer treatment will become even more complex and include multimodality therapies with chemotherapy, surgery, radiation and combined administration of various targeted antibodies. Therefore a continued emphasis is needed upon *in vitro* studies and animal models to further our understanding of sarcomagenesis and refine our use of these emerging technologies.

### References

1. Fearon ER, Vogelstein B. A genetic model for colorectal tumorigenesis. *Cell* 1990; 61: 759–67.
2. Ladanyi M, Bridge JA. Contribution of molecular genetic data to the classification of sarcomas. *Hum Pathol* 2000; 31: 532–8.
3. Rosen LS, Rosen PJ, Kabbinavar F, Mulay J, Mickey L, Hernandez JM, Brown J, Alexander C, Bello C, Cropp G, Kelsey S, Scigalla P. Phase I Experience with SU6668, a Novel Multiple Receptor Tyrosine Kinase Inhibitor in Patients with Advanced Malignancies. In Grunberg SM, ed. *American Society of Clinical Oncology* 20. San Francisco, ASCO Education, Science and Publications Department, 2001, 97A.
4. Casciato DA, Lowitz BB. *Manual of Clinical Oncology*. Boston, Little, Brown and Company, 1995.
5. DeVita VT. *Cancer Principles and Practice*. Philadelphia, Lippincott Williams and Wilkins, 2001.
6. Singer S. New diagnostic modalities in soft tissue sarcoma. *Semin Surg Oncol* 1999; 17: 11–22.
7. Kushner BH, LaQuaglia MP, Cheung NK, Kramer K, Hamelin AC, Gerald WL, Ladanyi M. Clinically critical impact of molecular genetic studies in pediatric solid tumors. *Med Pediatr Oncol* 1999; 33: 530–5.
8. Crozat A, Aman P, Mandahl N, Ron D. Fusion of CHOP to a novel RNA-binding protein in human myxoid liposarcoma. *Nature* 1993; 363: 640–4.
9. Clark J, Rocques PJ, Crew AJ, Gill S, Shipley J, Chan AM, Gusterson BA, Cooper CS. Identification of novel genes, SYT and SSX, involved in the t(X;18)(p11.2;q11.2) translocation found in human synovial sarcoma. *Nat Genet* 1994; 7: 502–8.
10. Kawai A, Woodruff J, Healey JH, Brennan MF, Antonescu CR, Ladanyi M. SYT-SSX gene fusion as a determinant of morphology and prognosis in synovial sarcoma. *N Engl J Med* 1998; 338: 153–60.
11. de Alava E, Antonescu CR, Panizo A, Leung D, Meyers PA, Huvos AG, Pardo-Mindan FJ, Healey JH, Ladanyi M. Prognostic impact of P53 status in Ewing sarcoma. *Cancer* 2000; 89: 783–92.
12. Skapek SX, Shaffer LG, Barker JA. Cytogenetics and the biological basis of sarcomas. *Curr Opin Oncol* 1998; 10: 318–25.
13. Aman P. Fusion genes in solid tumors. *Semin Cancer Biol* 1999; 9: 303–18.

14. Linehan DC, Bowne WB, Lewis JJ. Immunotherapeutic approaches to sarcoma. *Semin Surg Oncol* 1999; 17: 72–7.
15. Patel SR, Benjamin RS. New chemotherapeutic strategies for soft tissue sarcomas. *Semin Surg Oncol* 1999; 17: 47–51.
16. Tsuchiya H, Yamamoto N, Asada N, Terasaki T, Kanazawa Y, Takanaka T, Nishijima H, Tomita K. Caffeine-potentiated radiochemotherapy and function-saving surgery for high-grade soft tissue sarcoma. *Anticancer Res* 2000; 20: 2137–43.
17. Lewis JJ, Brennan MF. Soft tissue sarcomas. *Curr Probl Surg* 1996; 33: 817–72.
18. Tierney JF, Mosseri V, Stewart LA, Souhami RL, Parmar MK. Adjuvant chemotherapy for soft-tissue sarcoma: review and meta-analysis of the published results of randomised clinical trials. *Br J Cancer* 1995; 72: 469–75.
19. Parasuraman S, Rao BN, Bodner S, Cain A, Pratt CB, Merchant TE, Pappo AS. Clear cell sarcoma of soft tissues in children and young adults: the St. Jude Children's Research Hospital experience. *Pediatr Hematol Oncol* 1999; 16: 539–44.
20. Enzinger FM, Weiss SW. *Soft Tissue Tumors*. St. Louis, MO, Mosby-Year Book, Inc., 1995.
21. Coindre JM, Terrier P, Bui NB, Bonichon F, Collin F, Le Doussal V, Mandard AM, Vilain MO, Jacquemier J, Duplay H, Sastre X, Barlier C, Henry-Amar M, Mace-Lesech J, Contesso G. Prognostic factors in adult patients with locally controlled soft tissue sarcoma. A study of 546 patients from the French Federation of Cancer Centers Sarcoma Group. *J Clin Oncol* 1996; 14: 869–77.
22. van Geel AN, Pastorino U, Jauch KW, Judson IR, van Coevorden F, Buesa JM, Nielsen OS, Boudinet A, Tursz T, Schmitz PI. Surgical treatment of lung metastases: The European Organization for Research and Treatment of Cancer-Soft Tissue and Bone Sarcoma Group study of 255 patients. *Cancer* 1996; 77: 675–82.
23. Pisters PW, Harrison LB, Leung DH, Woodruff JM, Casper ES, Brennan MF. Long-term results of a prospective randomized trial of adjuvant brachytherapy in soft tissue sarcoma. *J Clin Oncol* 1996; 14: 859–68.
24. Pisters PW, Leung DH, Woodruff J, Shi W, Brennan MF. Analysis of prognostic factors in 1,041 patients with localized soft tissue sarcomas of the extremities. *J Clin Oncol* 1996; 14: 1679–89.
25. Tallman MS, Andersen JW, Schiffer CA, Appelbaum FR, Feusner JH, Ogden A, Shepherd L, Rowe JM, Francois C, Larson RS, Wiernik PH. Clinical description of 44 patients with acute promyelocytic leukemia who developed the retinoic acid syndrome. *Blood* 2000; 95: 90–5.
26. Tallman MS, Andersen JW, Schiffer CA, Appelbaum FR, Feusner JH, Ogden A, Shepherd L, Willman C, Bloomfield CD, Rowe JM, Wiernik PH. All-trans-retinoic acid in acute promyelocytic leukemia. *N Engl J Med* 1997; 337: 1021–8.
27. Joensuu H, Roberts PJ, Sarlomo-Rikala M, Andersson LC, Tervahartiala P, Tuveson D, Silberman S, Capdeville R, Dimitrijevic S, Druker B, Demetri GD. Effect of the tyrosine kinase inhibitor STI571 in a patient with a metastatic gastrointestinal stromal tumor. *N Engl J Med* 2001; 344: 1052–6.
28. Hirota S, Isozaki K, Moriyama Y, Hashimoto K, Nishida T, Ishiguro S, Kawano K, Hanada M, Kurata A, Takeda M, Muhammad Tunio G, Matsuzawa Y, Kanakura Y, Shinomura Y, Kitamura Y. Gain-of-function mutations of c-kit in human gastrointestinal stromal tumors. *Science* 1998; 279: 577–80.
29. Mackall C, Berzofsky J, Helman LJ. Targeting tumor specific translocations in sarcomas in pediatric patients for immunotherapy. *Clin Orthop* 2000: 25–31.
30. Goletz TJ, Mackall CL, Berzofsky JA, Helman LJ. Molecular alterations in pediatric sarcomas: potential targets for immunotherapy. *Sarcoma* 1998; 2: 77–87.
31. Bosilevac JM, Olsen RJ, Bridge JA, Hinrichs SH. Tumor cell viability in clear cell sarcoma requires DNA binding activity of the EWS/ATF1 fusion protein. *J Biol Chem* 1999; 274: 34811–8.
32. Brown AD, Lopez-Terrada D, Denny C, Lee KA. Promoters containing ATF-binding sites are de-regulated in cells that express the EWS/ATF1 oncogene. 1995; 10: 1749–1756.
33. May WA, Gishizky ML, Lessnick SL, Lunsford LB, Lewis BC, Delattre O, Zucman J, Thomas G, Denny CT. Ewing sarcoma 11;22 translocation produces a chimeric transcription factor that requires the DNA-binding domain encoded by FLI1 for transformation. *Proc Natl Acad Sci U S A* 1993; 90: 5752–6.
34. Kuroda M, Ishida T, Takanashi M, Satoh M, Machinami R, Watanabe T. Oncogenic transformation and inhibition of adipocytic conversion of preadipocytes by TLS/FUS-CHOP type II chimeric protein. *Am J Pathol* 1997; 151: 735–44.
35. Yi H, Fujimura Y, Ouchida M, Prasad DD, Rao VN, Reddy ES. Inhibition of apoptosis by normal and aberrant Fli-1 and erg proteins involved in human solid tumors and leukemias. *Oncogene* 1997; 14: 1259–68.
36. Lessnick SL, Braun BS, Denny CT, May WA. Multiple domains mediate transformation by the Ewing's sarcoma EWS/FLI-1 fusion gene. *Oncogene* 1995; 10: 423–31.
37. Bridge JA. Cytogenetics and experimental models. *Curr Opin Oncol* 1996; 8: 284–8.
38. Kim J, Pelletier J. Molecular genetics of chromosome translocations involving EWS and related family members. *Physiol Genomics* 1999; 1: 127–38.
39. Tobar A, Avigad S, Zoldan M, Mor C, Goshen Y, Zaizov R. Clinical relevance of molecular diagnosis in childhood rhabdomyosarcoma. *Diagn Mol Pathol* 2000; 9: 9–13.
40. Sassone-Corsi P. Coupling gene expression to cAMP signalling: role of CREB and CREM. *Int J Biochem Cell Biol* 1998; 30: 27–38.
41. Kovar H, Aryee D, Zoubek A. The Ewing family of tumors and the search for the Achilles' heel. *Curr Opin Oncol* 1999; 11: 275–84.
42. de Alava E, Gerald WL. Molecular biology of the Ewing's sarcoma/primitive neuroectodermal tumor family. *J Clin Oncol* 2000; 18: 204–13.
43. Ouchida M, Ohno T, Fujimura Y, Rao VN, Reddy ES. Loss of tumorigenicity of Ewing's sarcoma cells expressing antisense RNA to EWS-fusion transcripts. *Oncogene* 1995; 11: 1049–54.
44. Tanaka K, Iwakuma T, Harimaya K, Sato H, Iwamoto Y. EWS-Fli1 antisense oligodeoxynucleotide inhibits proliferation of human Ewing's sarcoma and primitive neuroectodermal tumor cells. *J Clin Invest* 1997; 99: 239–47.
45. Kovar H, Aryee DN, Jug G, Henockl C, Schemper M, Delattre O, Thomas G, Gadner H. EWS/FLI-1 antagonists induce growth inhibition of Ewing tumor cells in vitro. *Cell Growth Differ* 1996; 7: 429–37.
46. Toretsky JA, Connell Y, Neckers L, Bhat NK. Inhibition of EWS-FLI-1 fusion protein with antisense oligodeoxynucleotides. *J Neurooncol* 1997; 31: 9–16.
47. Lambert G, Bertrand JR, Fattal E, Subra F, Pinto-Alphandary H, Malvy C, Auclair C, Couvreur P.

EWS fli-1 antisense nanocapsules inhibits ewing sarcoma-related tumor in mice. *Biochem Biophys Res Commun* 2000; 279: 401–6.

48. Bridge J, Borek D, Neff J, Huntrakoon M. Chromosomal abnormalities in clear cell sarcoma. *Am J Clin Pathol* 1990; 93: 26–31.
49. Bridge J, Sreekantaiah C, Neff J, Sandberg A. Cytogenic findings in clear cell sarcoma of tendons and aponeuroses ('malignant melanoma of soft parts'). *Cancer Genet Cytogenet* 1991; 52: 101–106.
50. Gilchrist C, Orten D, Bosilevac J, Sanderson S, Hinrichs S. Transcriptional inhibition by a monoclonal antibody Fab fragment. *Antibody, Immunoconjugates and Radiopharmaceuticals* 1995; 8: 281–298.
51. Bosilevac JM, Gilchrist CA, Jankowski PE, Paul S, Rees AR, Hinrichs SH. Inhibition of activating transcription factor 1- and cAMP-responsive element-binding protein-activated transcription by an intracellular single chain Fv fragment. *J Biol Chem* 1998; 273: 16874–9.
52. Fredericks WJ, Ayyanathan K, Rauscher FJ, 3rd. Regulating the neoplastic phenotype using engineered transcriptional repressors. *Cancer Lett* 2001; 162 Suppl: S23–S32.
53. Bernasconi M, Remppis A, Fredericks WJ, Rauscher FJ, Schafer BW. Induction of apoptosis in rhabdomyosarcoma cells through down-regulation of PAX proteins. *Proc Natl Acad Sci U S A* 1996; 93: 13164–9.
54. Fredericks WJ, Ayyanathan K, Herlyn M, Friedman JR, Rauscher FJ. An engineered PAX3-KRAB transcriptional repressor inhibits the malignant phenotype of alveolar rhabdomyosarcoma cells harboring the endogenous PAX3-FKHR oncogene. *Mol Cell Biol* 2000; 20: 5019–31.
55. Hunter T, Karin M. The regulation of transcription by phosphorylation. *Cell* 1992; 70: 375–87.
56. Hunter T. Protein kinases and phosphatases: the yin and yang of protein phosphorylation and signaling. *Cell* 1995; 80: 225–36.
57. Girnita L, Wang M, Xie Y, Nilsson G, Dricu A, Wejde J, Larsson O. Inhibition of N-linked glycosylation down-regulates insulin-like growth factor-1 receptor at the cell surface and kills Ewing's sarcoma cells: therapeutic implications. *Anticancer Drug Des* 2000; 15: 67–72.
58. Wang M, Xie Y, Girnita L, Nilsson G, Dricu A, Wejde J, Larsson O. Regulatory role of mevalonate and N-linked glycosylation in proliferation and expression of the EWS/FLI-1 fusion protein in Ewing's sarcoma cells. *Exp Cell Res* 1999; 246: 38–46.
59. Toretsky JA, Thakar M, Eskenazi AE, Frantz CN. Phosphoinositide 3-hydroxide kinase blockade enhances apoptosis in the Ewing's sarcoma family of tumors. *Cancer Res* 1999; 59: 5745–50.
60. Stephenson J. Researchers buoyed by promise of targeted leukemia therapy. *Jama* 2000; 283: 317, 321.
61. Bennicelli JL, Barr FG. Genetics and the biologic basis of sarcomas. *Curr Opin Oncol* 1999; 11: 267–74.
62. Greco A, Roccato E, Miranda C, Cleris L, Formelli F, Pierotti MA. Growth-inhibitory effect of STI571 on cells transformed by the COL1A1/PDGFB rearrangement. *Int J Cancer* 2001; 92: 354–60.
63. Van Oosterom AT, Judson I, Verweij J, Di Paola E, van Glabbeke M, Dimitrijevic S, Nielsen O. STI571, an active drug in metastatic gastrointestinal stroma tumors (GIST), an EORTC phase I study. *American Society of Clinical Oncology* 1. San Francisco, CA, 2001, 1a.
64. Blanke CD, von Mehren M, Joensuu H, Roberts PJ, Eisenbert M, Heinrich M, Druker B, Tuveson D, Dimitrijevic S, Silberman SL, Demetri GD. Evaluation of the safety and efficacy of an oral molecularly-targeted therapy, STI571 in patients with unresectable or metastatic gastrointestinal stromal tumors (GIST) expressing D-KIT. *The American Society of Clinical Oncology* 1. San Francisco, CA, 2001, 1a.
65. Olsen RJ, Hinrichs SH. Phosphorylation of the EWS IQ domain regulates transcriptional activity of the EWS/ATF1 and EWS/FLI1 fusion proteins. *Oncogene* 2001; 20: 1756–64.
66. Lin PP, Brody RI, Hamelin AC, Bradner JE, Healey JH, Ladanyi M. Differential transactivation by alternative EWS-FLI1 fusion proteins correlates with clinical heterogeneity in Ewing's sarcoma. *Cancer Res* 1999; 59: 1428–32.
67. de Alava E, Kawai A, Healey JH, Fligman I, Meyers PA, Huvos AG, Gerald WL, Jhanwar SC, Argani P, Antonescu CR, Pardo-Mindan FJ, Ginsberg J, Womer R, Lawlor ER, Wunder J, Andrulis I, Sorensen PH, Barr FG, Ladanyi M. EWS-FLI1 fusion transcript structure is an independent determinant of prognosis in Ewing's sarcoma [published erratum appears in J Clin Oncol 1998 Aug;16(8):2895] [see comments]. *J Clin Oncol* 1998; 16: 1248–55.
68. Zoubek A, Dockhorn-Dworniczak B, Delattre O, Christiansen H, Niggli F, Gatterer-Menz I, Smith TL, Jurgens H, Gadner H, Kovar H. Does expression of different EWS chimeric transcripts define clinically distinct risk groups of Ewing tumor patients? *J Clin Oncol* 1996; 14: 1245–51.
69. Ladanyi M. Human Mesenchymal Tumors: Concepts in Pathology and Biology. In Marchesi VT, ed. *Experimental Biology* 15. Orlando, FASEB, 2001, S70.
70. Antelman D, Perry S, Hollingsworth R, Gregory RJ, Driscoll B, Fung YK, Bookstein R. Engineered mutants of pRB with improved growth suppression potential. *Oncogene* 1997; 15: 2855–66.
71. Caron de Fromentel C, Gruel N, Venot C, Debussche L, Conseiller E, Dureuil C, Teillaud JL, Tocque B, Bracco L. Restoration of transcriptional activity of p53 mutants in human tumour cells by intracellular expression of anti-p53 single chain Fv fragments. *Oncogene* 1999; 18: 551–7.
72. Karpeh MS, Brennan MF, Cance WG, Woodruff JM, Pollack D, Casper ES, Dudas ME, Latres E, Drobnjak M, Cordon-Cardo C. Altered patterns of retinoblastoma gene product expression in adult soft-tissue sarcomas. *Br J Cancer* 1995; 72: 986–91.
73. De Chiara A, T'Ang A, Triche TJ. Expression of the retinoblastoma susceptibility gene in childhood rhabdomyosarcomas. *J Natl Cancer Inst* 1993; 85: 152–7.
74. Toguchida J, Yamaguchi T, Ritchie B, Beauchamp RL, Dayton SH, Herrera GE, Yamamuro T, Kotoura Y, Sasaki MS, Little JB, *et al.* Mutation spectrum of the p53 gene in bone and soft tissue sarcomas. *Cancer Res* 1992; 52: 6194–9.
75. Wurl P, Meye A, Lautenschlager C, Schmidt H, Bache M, Kalthoff H, Schonfelder M, Rath FW, Taubert H. Clinical relevance of pRb and p53 co-overexpression in soft tissue sarcomas. *Cancer Lett* 1999; 139: 159–65.
76. Jiang H, Karnezis AN, Tao M, Guida PM, Zhu L. pRB and p107 have distinct effects when expressed in pRB-deficient tumor cells at physiologically relevant levels. *Oncogene* 2000; 19: 3878–87.
77. Latres E, Drobnjak M, Pollack D, Oliva MR, Ramos M, Karpeh M, Woodruff JM, Cordon-Cardo C. Chromosome 17 abnormalities and TP53 mutations in adult soft tissue sarcomas. *Am J Pathol* 1994; 145: 345–55.
78. Hainaut P, Soussi T, Shomer B, Hollstein M, Greenblatt M, Hovig E, Harris CC, Montesano R. Database of p53 gene somatic mutations in human tumors and cell lines: updated compilation and future prospects. *Nucleic Acids Res* 1997; 25: 151–7.
79. Li FP, Fraumeni JF, Jr. Soft-tissue sarcomas, breast cancer, and other neoplasms. A familial syndrome? *Ann Intern Med* 1969; 71: 747–52.
80. Hieken TJ, Das Gupta TK. Mutant p53 expression: a marker of diminished survival in well-differentiated soft tissue sarcoma. *Clin Cancer Res* 1996; 2: 1391–5.
81. Pollock JD, Rane SG. p21ras signaling is necessary but not sufficient to mediate neurotrophin induction of calcium channels in PC12 cells. *J Biol Chem* 1996; 271: 8008–14.
82. Taubert H, Meye A, Wurl P. Soft tissue sarcomas and p53 mutations. *Mol Med* 1998; 4: 365–72.
83. Pollock R, Lang A, Ge T, Sun D, Tan M, Yu D. Wild-type p53 and a p53 temperature-sensitive mutant suppress human soft tissue sarcoma by enhancing cell cycle control. *Clin Cancer Res* 1998; 4: 1985–94.
84. Milas M, Yu D, Lang A, Ge T, Feig B, El-Naggar AK, Pollock RE. Adenovirus-mediated p53 gene therapy inhibits human sarcoma tumorigenicity. *Cancer Gene Ther* 2000; 7: 422–9.
85. Watanabe T, Sullenger BA. Induction of wild-type p53 activity in human cancer cells by ribozymes that repair mutant p53 transcripts. *Proc Natl Acad Sci U S A* 2000; 97: 8490–4.
86. Zhang L, Yu D, Hu M, Xiong S, Lang A, Ellis LM, Pollock RE. Wild-type p53 suppresses angiogenesis in human leiomyosarcoma and synovial sarcoma by transcriptional suppression of vascular endothelial growth factor expression. *Cancer Res* 2000; 60: 3655–61.
87. Gibson AA, Harwood FG, Tillman DM, Houghton JA. Selective sensitization to DNA-damaging agents in a human rhabdomyosarcoma cell line with inducible wild-type p53 overexpression. *Clin Cancer Res* 1998; 4: 145–52.
88. Bougeret C, Virone-Oddos A, Adeline E, Lacroix F, Lefranc C, Ferrero L, Huet T. Cancer gene therapy mediated by CTS1, a p53 derivative: advantage over wild-type p53 in growth inhibition of human tumors overexpressing MDM2. *Cancer Gene Ther* 2000; 7: 789–98.
89. Schneider-Stock R, Walter H, Radig K, Rys J, Bosse A, Kuhnen C, Hoang-Vu C, Roessner A. MDM2 amplification and loss of heterozygosity at Rb and p53 genes: no simultaneous alterations in the oncogenesis of liposarcomas. *J Cancer Res Clin Oncol* 1998; 124: 532–40.
90. Barrios C, Castresana JS, Ruiz J, Kreicbergs A. Amplification of the c-myc proto-oncogene in soft tissue sarcomas. *Oncology* 1994; 51: 13–7.
91. Yap DB, Hsieh JK, Chan FS, Lu X. mdm2: a bridge over the two tumour suppressors, p53 and Rb. *Oncogene* 1999; 18: 7681–9.
92. Forus A, Florenes VA, Maelandsmo GM, Fodstad O, Myklebost O. The protooncogene CHOP/GADD153, involved in growth arrest and DNA damage response, is amplified in a subset of human sarcomas. *Cancer Genet Cytogenet* 1994; 78: 165–71.
93. Kobet E, Zeng X, Zhu Y, Keller D, Lu H. MDM2 inhibits p300-mediated p53 acetylation and activation by forming a ternary complex with the two proteins. *Proc Natl Acad Sci U S A* 2000; 97: 12547–52.
94. Chen J, Wu X, Lin J, Levine AJ. mdm-2 inhibits the G1 arrest and apoptosis functions of the p53 tumor suppressor protein. *Mol Cell Biol* 1996; 16: 2445–52.
95. Meye A, Wurl P, Bache M, Bartel F, Grunbaum U, Mansa-ard J, Schmidt H, Taubert H. Colony formation of soft tissue sarcoma cells is inhibited by lipid-mediated antisense oligodeoxynucleotides targeting the human mdm2 oncogene. *Cancer Lett* 2000; 149: 181–8.
96. Wang CY, Mayo MW, Baldwin AS. TNF- and cancer therapy-induced apoptosis: potentiation by inhibition of NF-kappaB. *Science* 1996; 274: 784–7.
97. Folkman J. Angiogenesis in cancer, vascular, rheumatoid and other disease. *Nat Med* 1995; 1: 27–31.
98. Yancopoulos GD, Davis S, Gale NW, Rudge JS, Wiegand SJ, Holash J. Vascular-specific growth factors and blood vessel formation. *Nature* 2000; 407: 242–8.
99. Folkman J. Tumor angiogenesis: therapeutic implications. *N Engl J Med* 1971; 285: 1182–6.
100. Folkman J. Angiogenesis and angiogenesis inhibition: an overview. *Exs* 1997; 79: 1–8.
101. Carmeliet P, Jain RK. Angiogenesis in cancer and other diseases. *Nature* 2000; 407: 249–57.
102. Vitaliti A, Wittmer M, Steiner R, Wyder L, Neri D, Klemenz R. Inhibition of tumor angiogenesis by a single-chain antibody directed against vascular endothelial growth factor. *Cancer Res* 2000; 60: 4311–4.
103. Brekken RA, Overholser JP, Stastny VA, Waltenberger J, Minna JD, Thorpe PE. Selective inhibition of vascular endothelial growth factor (VEGF) receptor 2 (KDR/Flk-1) activity by a monoclonal anti-VEGF antibody blocks tumor growth in mice. *Cancer Res* 2000; 60: 5117–24.
104. Gabrilovich DI, Ishida T, Nadaf S, Ohm JE, Carbone DP. Antibodies to vascular endothelial growth factor enhance the efficacy of cancer immunotherapy by improving endogenous dendritic cell function. *Clin Cancer Res* 1999; 5: 2963–70.
105. Mendel DB, Schreck RE, West DC, Li G, Strawn LM, Tanciongco SS, Vasile S, Shawver LK, Cherrington JM. The angiogenesis inhibitor SU5416 has long-lasting effects on vascular endothelial growth factor receptor phosphorylation and function. *Clin Cancer Res* 2000; 6: 4848–58.
106. Oku T, Tjuvajev JG, Miyagawa T, Sasajima T, Joshi A, Joshi R, Finn R, Claffey KP, Blasberg RG. Tumor growth modulation by sense and antisense vascular endothelial growth factor gene expression: effects on angiogenesis, vascular permeability, blood volume, blood flow, fluorodeoxyglucose uptake, and proliferation of human melanoma intracerebral xenografts. *Cancer Res* 1998; 58: 4185–92.
107. Nakashima T, Hudson JM, Clayman GL. Antisense inhibition of vascular endothelial growth factor in human head and neck squamous cell carcinoma. *Head Neck* 2000; 22: 483–8.
108. Eatock MM, Schatzlein A, Kaye SB. Tumour vasculature as a target for anticancer therapy. *Cancer Treat Rev* 2000; 26: 191–204.
109. Carter SK. Clinical strategy for the development of angiogenesis inhibitors. *Oncologist* 2000; 5: 51–4.
110. Kishi K, Petersen S, Petersen C, Hunter N, Mason K, Masferrer JL, Tofilon PJ, Milas L. Preferential enhancement of tumor radioresponse by a cyclooxygenase-2 inhibitor. *Cancer Res* 2000; 60: 1326–31.
111. Cahlin C, Gelin J, Delbro D, Lonnroth C, Doi C, Lundholm K. Effect of cyclooxygenase and nitric oxide synthase inhibitors on tumor growth in mouse tumor models with and without cancer cachexia related to prostanoids. *Cancer Res* 2000; 60: 1742–9.
112. Li P, Yuan M, Xia H. Experimental study of the therapeutic effects of an anti-endothelial cell monoclonal antibody BVE-1 for solid tumor xenograft in nude mice. *Zhonghua Zhong Liu Za Zhi* 1998; 20: 280–3.

113. Brooks PC, Montgomery AM, Rosenfeld M, Reisfeld RA, Hu T, Klier G, Cheresh DA. Integrin alpha v beta 3 antagonists promote tumor regression by inducing apoptosis of angiogenic blood vessels. *Cell* 1994; 79: 1157–64.
114. Gutheil JC, Campbell TN, Pierce PR, Watkins JD, Huse WD, Bodkin DJ, Cheresh DA. Targeted antiangiogenic therapy for cancer using Vitaxin: a humanized monoclonal antibody to the integrin alphavbeta3. *Clin Cancer Res* 2000; 6: 3056–61.
115. Moore PS, Chang Y. Detection of herpesvirus-like DNA sequences in Kaposi's sarcoma in patients with and without HIV infection. *N Engl J Med* 1995; 332: 1181–5.
116. Chang Y, Cesarman E, Pessin MS, Lee F, Culpepper J, Knowles DM, Moore PS. Identification of herpesvirus-like DNA sequences in AIDS-associated Kaposi's sarcoma. *Science* 1994; 266: 1865–9.
117. Bais C, Santomasso B, Coso O, Arvanitakis L, Raaka EG, Gutkind JS, Asch AS, Cesarman E, Gershengorn MC, Mesri EA, Gerhengorn MC. G-protein-coupled receptor of Kaposi's sarcoma-associated herpesvirus is a viral oncogene and angiogenesis activator. *Nature* 1998; 391: 86–9.
118. Montaner S, Sodhi A, Pece S, Mesri EA, Gutkind JS. The Kaposi's sarcoma-associated herpesvirus G protein-coupled receptor promotes endothelial cell survival through the activation of Akt/protein kinase B. *Cancer Res* 2001; 61: 2641–8.
119. Sodhi A, Montaner S, Patel V, Zohar M, Bais C, Mesri EA, Gutkind JS. The Kaposi's sarcoma-associated herpes virus G protein-coupled receptor up-regulates vascular endothelial growth factor expression and secretion through mitogen-activated protein kinase and p38 pathways acting on hypoxia-inducible factor 1alpha. *Cancer Res* 2000; 60: 4873–80.
120. Afford S, Randhawa S. Apoptosis. *Mol Pathol* 2000; 53: 55–63.
121. Wick MR. Immunohistology of neuroendocrine and neuroectodermal tumors. *Semin Diagn Pathol* 2000; 17: 194–203.
122. Dworzak MN, Fritsch G, Fleischer C, Printz D, Froschl G, Buchinger P, Mann G, Gadner H. CD99 (MIC2) expression in paediatric B-lineage leukaemia/lymphoma reflects maturation-associated patterns of normal B-lymphopoiesis. *Br J Haematol* 1999; 105: 690–5.
123. Hahn JH, Kim MK, Choi EY, Kim SH, Sohn HW, Ham DI, Chung DH, Kim TJ, Lee WJ, Park CK, Ree HJ, Park SH. CD99 (MIC2) regulates the LFA-1/ICAM-1-mediated adhesion of lymphocytes, and its gene encodes both positive and negative regulators of cellular adhesion. *J Immunol* 1997; 159: 2250–8.
124. Bernard G, Breittmayer JP, de Matteis M, Trampont P, Hofman P, Senik A, Bernard A. Apoptosis of immature thymocytes mediated by E2/CD99. *J Immunol* 1997; 158: 2543–50.
125. Scotlandi K, Baldini N, Cerisano V, Manara MC, Benini S, Serra M, Lollini PL, Nanni P, Nicoletti G, Bernard G, Bernard A, Picci P. CD99 engagement: an effective therapeutic strategy for Ewing tumors. *Cancer Res* 2000; 60: 5134–42.
126. Devaney K, Abbondanzo SL, Shekitka KM, Wolov RB, Sweet DE. MIC2 detection in tumors of bone and adjacent soft tissues. *Clin Orthop* 1995: 176–87.
127. Kovar H. Progress in the molecular biology of Ewing tumors. *Sarcoma* 1998; 2: 3–17.
128. Sohn HW, Choi EY, Kim SH, Lee IS, Chung DH, Sung UA, Hwang DH, Cho SS, Jun BH, Jang JJ, Chi JG, Park SH. Engagement of CD99 induces apoptosis through a calcineurin-independent pathway in Ewing's sarcoma cells. *Am J Pathol* 1998; 153: 1937–45.
129. Hasegawa T, Seki K, Ono K, Hirohashi S. Angiomatoid (malignant) fibrous histiocytoma: a peculiar low-grade tumor showing immunophenotypic heterogeneity and ultrastructural variations. *Pathol Int* 2000; 50: 731–8.
130. Kim DH, Sohn JH, Lee MC, Lee G, Yoon GS, Hashimoto H, Sonobe H, Ro JY. Primary synovial sarcoma of the kidney. *Am J Surg Pathol* 2000; 24: 1097–104.
131. Aramburu-Gonzalez JA, Rodriguez-Justo M, Jimenez-Reyes J, Santonja C. A case of soft tissue mesenchymal chondrosarcoma metastatic to skin, clinically mimicking keratoacanthoma. *Am J Dermatopathol* 1999; 21: 392–4.
132. Fisher C. Synovial sarcoma. *Ann Diagn Pathol* 1998; 2: 401–21.
133. Ordi J, de Alava E, Torne A, Mellado B, Pardo-Mindan J, Iglesias X, Cardesa A. Intraabdominal desmoplastic small round cell tumor with EWS/ERG fusion transcript. *Am J Surg Pathol* 1998; 22: 1026–32.
134. Itoh N, Yonehara S, Ishii A, Yonehara M, Mizushima S, Sameshima M, Hase A, Seto Y, Nagata S. The polypeptide encoded by the cDNA for human cell surface antigen Fas can mediate apoptosis. *Cell* 1991; 66: 233–43.
135. Nagata S, Golstein P. The Fas death factor. *Science* 1995; 267: 1449–56.
136. Kontny HU, Lehrnbecher TM, Chanock SJ, Mackall CL. Simultaneous expression of Fas and nonfunctional Fas ligand in Ewing's sarcoma. *Cancer Res* 1998; 58: 5842–9.
137. Hamada T, Komiya S, Yano H, Zenmyo M, Hiraoka K, Inoue A, Morimatsu M. Modulation of fas-mediated apoptosis in osteosarcoma cell lines. *Int J Oncol* 1999; 15: 1125–31.
138. Springer ML, Kraft PE, Blau HM. Inhibition of solid tumor growth by Fas ligand-expressing myoblasts. *Somat Cell Mol Genet* 1998; 24: 281–9.
139. Aoki K, Akyurek LM, San H, Leung K, Parmacek MS, Nabel EG, Nabel GJ. Restricted expression of an adenoviral vector encoding Fas ligand (CD95L) enhances safety for cancer gene therapy. *Mol Ther* 2000; 1: 555–65.
140. Takebayashi H, Oida H, Fujisawa K, Yamaguchi M, Hikida T, Fukumoto M, Narumiya S, Kakizuka A. Hormone-induced apoptosis by Fas-nuclear receptor fusion proteins: novel biological tools for controlling apoptosis in vivo. *Cancer Res* 1996; 56: 4164–70.
141. Chen F, Castranova V, Shi X, Demers LM. New insights into the role of nuclear factor-kappaB, a ubiquitous transcription factor in the initiation of diseases. *Clin Chem* 1999; 45: 7–17.
142. Sonenshein GE. Rel/NF-kappa B transcription factors and the control of apoptosis. *Semin Cancer Biol* 1997; 8: 113–9.
143. Higgins KA, Perez JR, Coleman TA, Dorshkind K, McComas WA, Sarmiento UM, Rosen CA, Narayanan R. Antisense inhibition of the p65 subunit of NF-kappa B blocks tumorigenicity and causes tumor regression. *Proc Natl Acad Sci U S A* 1993; 90: 9901–5.
144. Feig BW, Lu X, Hunt KK, Shan Q, Yu D, Pollock R, Chiao P. Inhibition of the transcription factor nuclear factor-kappa B by adenoviral-mediated expression of I kappa B alpha M results in tumor cell death. *Surgery* 1999; 126: 399–405.

145. Black AR, Azizkhan-Clifford J. Regulation of E2F: a family of transcription factors involved in proliferation control. *Gene* 1999; 237: 281–302.
146. Hunt KK, Liu TJ, Feig B. Induction of apoptosis in a leiomyosarcoma cell line by adenoviral-mediated over-expression of the transcription factor E2F-1. *Sarcoma J* 1997; 1: 194–195.
147. Hunt KK, Feig BW. Preclinical experimental therapeutic approaches in soft tissue sarcoma. *Semin Surg Oncol* 1999; 17: 78–82.
148. Hetrakul N, Abramian A, Liu T. Growth inhibition of leiomyosarcoma in vivo using an adenovirus overexpressing the transcription factor E2F-1. *Sarcoma J* 1999; 3: 49–51.
149. Goldfarb M. Functions of fibroblast growth factors in vertebrate development. *Cytokine Growth Factor Rev* 1996; 7: 311–25.
150. Sturla LM, Westwood G, Selby PJ, Lewis IJ, Burchill SA. Induction of cell death by basic fibroblast growth factor in Ewing's sarcoma. *Cancer Res* 2000; 60: 6160–70.
151. Villunger A, Strasser A. Does death receptor signaling play a role in tumorigenesis and cancer therapy? *Oncol Res* 1998; 10: 541–50.
152. Yuyama Y, Tsujimoto M, Fujimoto Y, Oku N. Potential usage of thermosensitive liposomes for site-specific delivery of cytokines. *Cancer Lett* 2000; 155: 71–7.
153. van Valen F, Fulda S, Truckenbrod B, Eckervogt V, Sonnemann J, Hillmann A, Rodl R, Hoffmann C, Winkelmann W, Schafer L, Dockhorn-Dworniczak B, Wessel T, Boos J, Debatin KM, Jurgens H. Apoptotic responsiveness of the Ewing's sarcoma family of tumours to tumour necrosis factor-related apoptosis-inducing ligand (TRAIL). *Int J Cancer* 2000; 88: 252–9.
154. Eggermont AM, Schraffordt Koops H, Klausner JM, Kroon BB, Schlag PM, Lienard D, van Geel AN, Hoekstra HJ, Meller I, Nieweg OE, Kettelhack C, Ben-Ari G, Pector JC, Lejeune FJ. Isolated limb perfusion with tumor necrosis factor and melphalan for limb salvage in 186 patients with locally advanced soft tissue extremity sarcomas. The cumulative multicenter European experience. *Ann Surg* 1996; 224: 756–64; discussion 764–5.
155. Shand N, Weber F, Mariani L, Bernstein M, Gianella-Borradori A, Long Z, Sorensen AG, Barbier N. A phase 1–2 clinical trial of gene therapy for recurrent glioblastoma multiforme by tumor transduction with the herpes simplex thymidine kinase gene followed by ganciclovir. GLI328 European-Canadian Study Group. *Hum Gene Ther* 1999; 10: 2325–35.
156. Eck SL, Alavi JB, Alavi A, Davis A, Hackney D, Judy K, Mollman J, Phillips PC, Wheeldon EB, Wilson JM. Treatment of advanced CNS malignancies with the recombinant adenovirus H5.010RSVTK: a phase I trial. *Hum Gene Ther* 1996; 7: 1465–82.
157. Uchida A, Seto M, Hashimoto N, Araki N. Molecular diagnosis and gene therapy in musculoskeletal tumors. *J Orthop Sci* 2000; 5: 418–23.
158. Candotti F, Agbaria R, Mullen CA, Touraine R, Balzarini J, Johns DG, Blaese RM. Use of a herpes thymidine kinase/neomycin phosphotransferase chimeric gene for metabolic suicide gene transfer. *Cancer Gene Ther* 2000; 7: 574–80.
159. Gazit G, Hung G, Chen X, Anderson WF, Lee AS. Use of the glucose starvation-inducible glucose-regulated protein 78 promoter in suicide gene therapy of murine fibrosarcoma. *Cancer Res* 1999; 59: 3100–6.
160. Marconi P, Tamura M, Moriuchi S, Krisky DM, Niranjan A, Goins WF, Cohen JB, Glorioso JC. Connexin 43-enhanced suicide gene therapy using herpesviral vectors. *Mol Ther* 2000; 1: 71–81.
161. Ko SC, Cheon J, Kao C, Gotoh A, Shirakawa T, Sikes RA, Karsenty G, Chung LW. Osteocalcin promoter-based toxic gene therapy for the treatment of osteosarcoma in experimental models. *Cancer Res* 1996; 56: 4614–9.
162. Massuda ES, Dunphy EJ, Redman RA, Schreiber JJ, Nauta LE, Barr FG, Maxwell IH, Cripe TP. Regulated expression of the diphtheria toxin A chain by a tumor-specific chimeric transcription factor results in selective toxicity for alveolar rhabdomyosarcoma cells. *Proc Natl Acad Sci U S A* 1997; 94: 14701–6.
163. Mizuguchi H, Nakanishi T, Nakanishi M, Nakagawa T, Nakagawa S, Mayumi T. Intratumor administration of fusogenic liposomes containing fragment A of diphtheria toxin suppresses tumor growth. *Cancer Lett* 1996; 100: 63–9.
164. Mizuguchi H, Nakanishi M, Nakanishi T, Nakagawa T, Nakagawa S, Mayumi T. Application of fusogenic liposomes containing fragment A of diphtheria toxin to cancer therapy. *Br J Cancer* 1996; 73: 472–6.
165. Danks MK, Morton CL, Pawlik CA, Potter PM. Overexpression of a rabbit liver carboxylesterase sensitizes human tumor cells to CPT-11. *Cancer Res* 1998; 58: 20–2.
166. Pawlik CA, Iyengar RV, Krull EJ, Mason SE, Khanna R, Harris LC, Potter PM, Danks MK, Guichard SM. Use of the ornithine decarboxylase promoter to achieve N-MYC-mediated overexpression of a rabbit carboxylesterase to sensitize neuroblastoma cells to CPT-11. *Mol Ther* 2000; 1: 457–63.
167. Goldman JM, Melo JV. Targeting the BCR-ABL tyrosine kinase in chronic myeloid leukemia. *N Engl J Med* 2001; 344: 1084–6.
168. Roth JA. Restoration of tumour suppressor gene expression for cancer. *Forum (Genova)* 1998; 8: 368–76.
169. Wei G, Antonescu CR, de Alava E, Leung D, Huvos AG, Meyers PA, Healey JH, Ladanyi M. Prognostic impact of INK4A deletion in Ewing sarcoma. *Cancer* 2000; 89: 793–9.
170. Roth JA, Swisher SG, Meyn RE. p53 tumor suppressor gene therapy for cancer. *Oncology (Huntingt)* 1999; 13: 148–54.
171. Uherek C, Wels W. DNA-carrier proteins for targeted gene delivery. *Adv Drug Deliv Rev* 2000; 44: 153–66.
172. Kohler G, Milstein C. Continuous cultures of fused cells secreting antibody of predefined specificity. *Nature* 1975; 256: 495–7.
173. Baselga J. Clinical trials of Herceptin(trastuzumab). *Eur J Cancer* 2001; 37 Suppl 1: S18–24.
174. Ehrlich P. The Nobel lectures in immunology. The Nobel prize for physiology or medicine, 1908, awarded to Elie Metchnikoff & Paul Ehrlich "in recognition of their work on immunity & quot. *Scand J Immunol* 1990; 31: 1–13.
175. Eisenhauer EA. From the molecule to the clinic–inhibiting HER2 to treat breast cancer. *N Engl J Med* 2001; 344: 841–2.
176. Kottaridis PD, Milligan DW, Chopra R, Chakraverty RK, Chakrabarti S, Robinson S, Peggs K, Verfuerth S, Pettengell R, Marsh JC, Schey S, Mahendra P, Morgan GJ, Hale G, Waldmann H, de Elvira MC, Williams CD, Devereux S, Linch DC, Goldstone AH, Mackinnon S. In vivo CAMPATH-1H prevents graft-versus-host disease following nonmyeloablative stem cell transplantation. *Blood* 2000; 96: 2419–25.
177. Coiffier B, Haioun C, Ketterer N, Engert A, Tilly H, Ma D, Johnson P, Lister A, Feuring-Buske M, Radford JA, Capdeville R, Diehl V, Reyes F. Rituximab

(anti-CD20 monoclonal antibody) for the treatment of patients with relapsing or refractory aggressive lymphoma: a multicenter phase II study. *Blood* 1998; 92: 1927–32.

178. Saltz L, Rubin M, Hochster H, Tchekmeydian S, Waksal H, Needle M, LoBuglio A. Cetuximab (IMC-C225) Plus Irinotecan (CPT-11) is Active in CPT-11-Refractory Colorectal Cancer (CRC) that Expresses Epidermanl Growth Factor Receptor (EGFR). In Grunberg SM, ed. *American Society of Clinical Oncology*. San Francisco, ASCO Education, Science and Publications Department, 2001, 3a.
179. Niculescu-Duvaz I. Technology evaluation: gemtuzumab ozogamicin, Celltech Group. *Curr Opin Mol Ther* 2000; 2: 691–6.
180. Davies AJ, Radford JA, Britton K, Howell S, Deakin DP, Micallef IN, Foley R, Barlow R, Carrington BM, Lawrence JA, Kegley P, Morris A, Owen S, Vinnicombe S, Harris M, Norton A, Lister TA, Rohatiner AZ. A Phase II study of Bexxar (Tositumomab and I-131 Tositumomab) for Patioents at 1st or 2nd Recurrence of B-Cell Non Hodgkin's Lymphoma. In Grunberg SM, ed. *American Society of Clinical Oncologists*. San Francisco, ASCO Education, Science and Publications, 2001, 1142.
181. Emmanoulidies C, Witzig T, Gordon L, Wiseman G, Bartlett N, Murray J, Multani P. Zevalin Radioimmunotherapy (RIT) is Safe and Effective in Geriatric Patients with Low Grade Follicular or CD20+ Transformed (LG/F/T) Non-Hodgkin's Lymphoma. In Grunberg SM, ed. *American Society of Clinical Oncology*. San Francisco, ASCO Education, Science and Publications, 2001, 1143.
182. Ward ES, Gussow D, Griffiths AD, Jones PT, Winter G. Binding activities of a repertoire of single immunoglobulin variable domains secreted from Escherichia coli. *Nature* 1989; 341: 544–6.
183. Colcher D, Minelli MF, Roselli M, Muraro R, Simpson-Milenic D, Schlom J. Radioimmunolocalization of human carcinoma xenografts with B72.3 second generation monoclonal antibodies. *Cancer Res* 1988; 48: 4597–603.
184. Yokota T, Milenic DE, Whitlow M, Schlom J. Rapid tumor penetration of a single-chain Fv and comparison with other immunoglobulin forms. *Cancer Res* 1992; 52: 3402–8.
185. Rodwell JD. Engineering monoclonal antibodies. *Nature* 1989; 342: 99–100.
186. Goel A, Colcher D, Baranowska-Kortylewicz J, Augustine S, Booth BJ, Pavlinkova G, Batra SK. Genetically engineered tetravalent single-chain Fv of the pancarcinoma monoclonal antibody CC49: improved biodistribution and potential for therapeutic application. *Cancer Res* 2000; 60: 6964–71.
187. Worn A, Pluckthun A. Stability Engineering of Antibody Single-chain Fv Fragments. *J Mol Biol* 2001; 305: 989–1010.
188. Adjuvant chemotherapy for localised resectable soft-tissue sarcoma of adults: meta-analysis of individual data. Sarcoma Meta-analysis Collaboration. *Lancet* 1997; 350: 1647–54.
189. Gortzak E, Azzarelli A, Buesa J, Bramwell VH, van Coevorden F, van Geel AN, Ezzat A, Santoro A, Oosterhuis JW, van Glabbeke M, Kirkpatrick A, Verweij J. A randomised phase II study on neo-adjuvant chemotherapy for 'high-risk' adult soft-tissue sarcoma. *Eur J. Cancer* 2001; 37: 1096–103.
